# Community Ownership in Primary Health Care—Managing the Intangible

**DOI:** 10.9745/GHSP-D-20-00427

**Published:** 2020-09-30

**Authors:** Eric Sarriot, Ali Nashat Shaar

**Affiliations:** a Save the Children, Washington DC, USA.; b Palestinian Child Institute, An-Najah National University, Nablus, Palestine.

## Abstract

Although enduringly intangible, community ownership is foundational to primary health care. This intangibility is a reminder of what programs can and should do (create space for dialogue, question their own choices, expand diversity in stakeholder voices making sense of program-induced changes, including through evaluation) and what they cannot do (manage someone else’s ownership).

## Key Messages

- The concept of community ownership in primary health care has a long history but remains challenged in terms of definition, measurement, and differences of perspective from practitioners on a gradient between utilitarianism and empowerment. It continues to be somewhat intangible.
- Although a universal definition across time and contexts may be illusory, contextual appreciation of its dynamic evolution under programmatic influences—for different stakeholders with diverse agendas—is accessible to evaluation and learning.
- No one can “manage” someone else’s ownership, but programs can reject hubris and tokenism by intentionally questioning their unavoidable impact on community ownership and whether they foster it through meaningful dialogue and “sense-making” with local stakeholders.

See related article by Fontanet et al.

In this issue of GHSP, Fontanet et al.^1^ invite us to return to a concept that has existed since early discussions of community medicine^2^ and primary health care^3^: community ownership in health. Many of us who work in global health have felt and seen the excitement and sense of possibility when communities took charge, made a project “their own,” innovated to find contextual solutions, and generated energy and hope in addition to buy-in for a lifesaving or health-promoting intervention. In 1992, one of this article’s authors witnessed how heavy rains had damaged a clinic serving the poor population of Jiftlik in the Jordan valley. Without institutional funds to rehabilitate the structure, the village residents felt a sense of ownership and accountability and restored the clinic themselves, and this clinic is still providing services in 2020. The literature is rich with case studies like this.^4^^–^^6^

As critical as community ownership is—and even foundational for many—it also appears to remain somewhat intangible, possibly impractical for some, and certainly complex for all. We consider some of the reasons for this quandary.

## DEFINING COMMUNITY OWNERSHIP

The first stumbling block with community ownership is definitional. This naturally starts with, “what is community really?” This question is followed by—as we generally discuss social processes writ large rather than physical assets^7^—“what is ownership?” We will satisfy ourselves for now with the idea that a community can be a geographically and demographically defined group of people, a network of people with a common agenda or challenge (illness), and/or most likely a combination of both of these, which creates the possibility of being **in** a community but **outside** of important social relationships.

Fontanet et al.^1^ remind us of the looseness of the concept of community ownership and frame it first under the Paris Declaration of Aid Effectiveness^8^; community ownership would fit with country ownership, albeit on a different, more local scale. (Oxfam and Save the Children, for their part, see a shift in emphasis from community to country as “a more state-centric form of ownership.”^9^)

Community ownership is sometimes defined through **requirements** for ownership, including capacity, empowerment, leadership, value found in the provision of a service, aspirations, and participation, or through **consequences** of ownership, including participation (again), financial commitment, contributions, and organization membership.^10^^–^^15^ These definitions can sometimes appear tautological—that ownership is defined by the fact of owning or institutionalizing a process or a goal. The literature associates ownership with sustainability of activities and outcomes, a means to achieve cultural adaptation for effective intervention models and to build problem-solving capacity.^10^^,^^12^^,^^16^ Ownership can be described as a requirement to build community capacity in a health promotion effort, yet capacity can be presented as a requirement of ownership.^10^ Whichever way the causal link is created, it is presented on the path to effective and sustainable health interventions. Countless evaluation reports have also associated failure of achievement and sustainability to the lack of community ownership generated by external projects. In the past, the concept has also been associated to financial contributions by communities,^17^ something critically revised through the universal health coverage agenda.

Much like the concept of participation, ownership lives in the tension between utilitarianism and empowerment,^18^ bridging over to human rights, democratic, and humanist perspectives on development processes. The Ottawa Charter for Health Promotion encouraged a process for enabling communities to increase control over and improve health and notably stated^19^:

*Health promotion works through concrete and effective community action in setting priorities, making decisions, planning strategies and implementing them to achieve better health. At the heart of this process is the empowerment of communities—their ownership and control of their own endeavors and destinies.*

Advancing community ownership faces at least 3 other challenges.

## IDEALISTIC FRAMING

Although we support and believe in the Ottawa Charter’s vision of seeking to increase people’s control over their own health, we must also acknowledge that calls for ownership and “full participation” (as in the recent Astana statement^20^) sometimes contain an element of idealism that pragmatists can occasionally point out with a wink or with cynicism in the face of harsh “field” realities. Community members may in fact be satisfied sometimes by simply being clients of health services. Demands for social accountability surge when quality, equity, responsiveness, and access conditions are not met. But when they are, people might satisfy themselves with utilizing, rather than owning, a service.

Indeed, public health problems are defined in a context, and these “problems-in-context” demand specific solution configurations, not all of which require the same level of social engagement. People responding to an acute threat might not perceive ownership as an immediate priority. Of course, the global health community had to rapidly re-discover the importance of building a response **with** communities in the Ebola emergency and efforts to eradicate polio.^21^^,^^22^ The current global challenges with vaccine acceptance and the coronavirus disease (COVID-19) situation^23^ are also signaling that some form of ownership is required for scale, sustainability, and impact of interventions. Still, we must also acknowledge that many short-term bets can be won with money and energy invested in proximal determinants of health. Ownership is critical but may be a distal determinant of success. We undermine our own advocacy if we appear to take for granted the value of technicity, policy, and organization in solving health challenges and present ownership in absolute terms.

We undermine our own advocacy if we appear to take for granted the value of technicity, policy, and organization in solving health challenges and present ownership in absolute terms.

## UNDERAPPRECIATION FOR THE INHERENT THREAT TO OWNERSHIP FROM EXTERNAL PROJECTS

Why are we asking about ownership ultimately? Because although they are always well-intended, not infrequently effective, and sometimes sustainable, our external projects inherently displace power and ownership from “natural” social systems (if there is such a thing). We punctuate an equilibrium, if not of ownership, at least of acceptance or resignation to a social baseline, but unless some new equilibrium of ownership is found between diverse stakeholders, the system will be attracted back to its baseline or some other suboptimal state.

Ignoring this tension poses a great risk of hubris. We know the stereotype: experts can come and “give messages,” tell people what the evidence says, and incentivize them to follow their plan, while failing to listen honestly and with respect to the local and community-appropriate ideas for adaptation of the approaches. White elephants are built. Without being a cynic, simply having self-satisfaction with giving token respect for the value of community ownership or coopting can lead to asking the wrong questions, in other words, having a poor definition of what problems really need to be addressed in context. Policy makers close a market to create social distancing; populations protest because they weigh differently an epidemiological risk against the necessity of feeding their family; the market reopens, but no effective community-owned risk reduction solution has been developed.

Although the concern about projects’ displacement of ownership may have been born out of an evolution of international programs away from colonialism, “do-gooding,” and hubris, it also applies to any national or regional program trying to reach remote, poor, minority, or neglected areas. Displacement of ownership is not an international development problem; it is a universal central-to-local (resource rich to resource poor) development problem. And while “we” question “their” ownership, we are rarely fully accountable for what role and agency we choose to keep to ourselves as we transition.^24^

## MEASUREMENT FOR PROGRAMS

We already mentioned different dimensions through which ownership has been framed. Efforts at measurement naturally must also be multidimensional,^9^ but this is not the greatest measurement challenge. Research may be able to draw conclusions from a distance on the ownership demonstrated by various communities and stakeholders, but program evaluation—seeking to assess what allows or hinders ownership during implementation—must be carried out **with** the stakeholders or else be meaningless.

As is the case for assessing institutional capacity, assessing or measuring ownership requires that the “owners” at least acquiesce to the process. A thought experiment can make the point. How would our employers or neighbors react to an outsider knocking on their virtual door to measure their ownership of a stated goal? While accepting to step on the scale does not influence the weight that will be posted on the scale, the measurement of a community’s ownership has community prerequisites in terms of buy-in and boundary decisions (who is the community and who is asking the question?). The prerequisites for measuring ownership are not independent of the ownership variable. It is noteworthy that Fontanet et al. allowed different stakeholders to define their ownership differently. Elements of subjectivity seem unavoidable—not something typically desired in project performance management.

The prerequisites for measuring ownership are not independent of the ownership variable.

This subjectivity comes with management challenges. Projects try to manage by results and give evidence for achievements. We develop indicators that are as objective and reliable as possible. But when it comes to measuring changes in a social system, our log frames and theories of change are challenged to capture the interaction between our programs and social dynamics over time.^25^ We say that we “cannot manage it if we cannot measure it,” but given the nature of the question, can we ever manage the ownership of someone else? Then, what are we trying to measure, who should be doing the measurement, and over what timeframe, if ownership evolves on a different timeline than service outputs?

Last and not least, ownership in a complex social system is always changing (dynamic) and can be affected by small changes in interpersonal relationships, services, or operational rules. A new equilibrium between stakeholders comes with new rules and boundaries, and questions may be raised about the ownership allowed for newcomers.^11^ The stakeholders of community ownership will change, their relationships will change, their perspectives will evolve, as shown by Fontanet et al. over just a 24-month period.

This leaves us with a series of limitations:

- We should assess our impact on community ownership, but our measurement is likely to be subjective and flawed.
- We want to be accountable for progress, but community ownership is precisely about things that we must let go of.
- We should be concerned about community ownership, but we still cannot totally define it. Its local definition depends on who sits around the table. It may change and change substantially based on small evolutions of the problem-in-context.

Should we just abandon all hope? Perhaps not.

## CONCLUSION

Social scientists will continue to enrich our understanding by dissecting ownership for different problems and contexts. The measurement challenge may be like that of social capital, for which operational measures can be defined in different contexts, even if a set of universal measures for all contexts may remain out of reach.^26^ Fontanet et al.^1^ interestingly circumvent some of the challenges by exploring with qualitative rigor the **perceptions** of ownership, providing substance to the concept from stakeholders, who have different but compatible definitions of what ownership is to them. The intangible is not made totally tangible, but the local meaning for stakeholders provides guidance to continue developing a program. An-other role of research may thus be to provide substance for advocacy and to challenge approaches that deny agency to marginalized communities.

Not all programs have access to strong research capability. However, they can use monitoring, learning, evaluation, and accountability tools to limit disrupting ownership or even to foster it. Promoting community ownership and learning about its development may be more akin to generating new social equilibria than planning for the delivery of a discrete outcome. It demands genuine interactions, creating enabling conditions and spaces for incremental changes, and building shared values. These ideas are not far from the concept of “harnessing complexity” in complex social and institutional systems.^27^ It quite possibly will require monitoring “us”—how we use our money, power, and time, and maybe addressing more critically when we must act and when we must choose to use restraint—as much as measuring “their” ownership. Sustainability-conscious public health practitioners, whether national or international, may not need to worry about precisely measuring the state of community ownership, but to focus more on which agents of the local system are taking agency, how much, and how diverse voices give meaning to tangible changes and intangible perceptions about structures, services, actions, relationships, and values.

If we are intent on finding viable long-term solutions to primary health care challenges with a view of Sustainable Development,^28^ transition, and the “journey to self-reliance,”^29^ the greatest mistake may be failing to critically engage in questioning our projects’ effects on community ownership and to mistrust the ability of communities to be agents of change.

As messy as it may be.
